# Sexual Orientation and Suicide Risk: Examining the Contributions of Hopelessness, Life Satisfaction, and Spirituality

**DOI:** 10.3390/bs16030406

**Published:** 2026-03-10

**Authors:** Félix Arbinaga, Jara Durán-Andrada, Cristina Fuentes-Méndez, Manuel Flores-Pérez, Nehemías Romero-Pérez, Lidia Torres-Rosado, Miriam Bernal-López

**Affiliations:** 1 Department of Clinical and Experimental Psychology, Faculty of Education, Psychology and Sports Science, University of Huelva, 21007 Huelva, Spain; cfuentesmendez@gmail.com (C.F.-M.); manuel.fperez@alu.uhu.es (M.F.-P.); nehemias.romero@dpces.uhu.es (N.R.-P.); lidia.torres@dpces.uhu.es (L.T.-R.); 2 Center for Research in Contemporary Thought and Innovation for Social Development (COIDESO), University of Huelva, 21007 Huelva, Spain; 3 Department of Social, Development and Educational Psychology, Faculty of Education, Psychology and Sports Science, University of Huelva, 21007 Huelva, Spain; miriam.bernal@dpces.uhu.es

**Keywords:** suicide, sexual orientation, hopelessness, life satisfaction, spirituality, religiosity, gay, lesbian, heterosexuality, bisexuality

## Abstract

Suicidal behaviors constitute a major global public health problem, with sexual minority groups showing a higher risk of engaging in such behaviors. This study aimed to analyze the influence of hopelessness, life satisfaction, and spirituality on suicide risk according to self-reported sexual orientation. A total of 532 individuals participated (M = 31.15 years, SD = 12.002). Of these, 39.8% identified as heterosexual, 34.2% as gay or lesbian individuals, and 25.9% as bisexual. Participants were assessed using the Plutchik Suicide Risk Scale, Beck’s Hopelessness Scale, the Beliefs and Values Scale, and Diener’s Satisfaction with Life Scale. The results indicate that 52.9% of bisexual participants and 41.2% of gay and lesbian participants presented a high suicide risk, compared with 15.6% of heterosexual participants. Individuals with a high suicide risk reported higher levels of hopelessness (*p* < 0.001), lower levels of life satisfaction (*p* < 0.001), and similar levels of spirituality. The proportion of variance explained in suicide risk was 42.8% among bisexual participants, 34.2% among gay and lesbian participants, and 29.9% among heterosexual participants. Hopelessness predicted a similar proportion of across groups (β = 0.446 in heterosexuals, β = 0.447 in gays and lesbians, and β = 0.457 in bisexuals). Life satisfaction showed a protective predictive effect, with β = −0.241 in bisexual participants, followed by gay and lesbian participants (β = −0.186) and heterosexual participants (β = −0.137). Spirituality was significant only among gay and lesbian participants (β = 0.133) and bisexual participants (β = 0.214). Sexual minority groups exhibited a higher risk of suicide, with life satisfaction—but not spirituality—acting as a protective factor.

## 1. Introduction

Suicidal behaviors constitute a problem whose consequences extend beyond personal, familial, social, and economic spheres (WHO, 2021b). Suicide has been reported as one of the leading causes of death among adolescents and young adults (Glenn et al., 2020; Mortier et al., 2017). Current estimates indicate that nearly 800,000 deaths worldwide occur by suicide each year (Abdulai, 2020; WHO, 2025), accounting for approximately 1% of all deaths globally (WHO, 2021a). For each death by suicide, an estimated 115 individuals are affected, and one in five individuals report having experienced a significant disruption in their lives as a consequence (Baca-Garcia et al., 2011; Sandler, 2018).

In this context, suicide among sexual minority individuals (e.g., gay, lesbian, bisexual) represents a significant public health problem (Blosnich, 2022). Compared with heterosexual individuals, sexual minority populations have approximately a 2.5- to 5-fold greater lifetime risk of attempting suicide (Hottes et al., 2016; Salway et al., 2019) and show higher suicide rates overall (Cochran & Mays, 2015; Erlangsen et al., 2020; Lynch et al., 2020; Mathy et al., 2011). A similar pattern has been observed among young gay, lesbian and bisexual populations, who were more than twice as likely as their heterosexual peers to have considered attempting suicide in the past year (31% vs. 14%) (District of Columbia Public Schools, 2007).

Regarding the relationship between sexual orientation and suicidal ideation, findings indicate that significant differences emerge between heterosexual and gay and lesbian individuals, as well as between heterosexual and bisexual individuals (Avendaño-Prieto et al., 2019; Mozumder et al., 2023). In other populations, such as transgender individuals, the prevalence of suicidal ideation has been reported to range from 50% to 75% (Nahata et al., 2017; Sorbara et al., 2020), with suicide attempt rates reaching 30% or higher (Nahata et al., 2017).

Similarly, the RaRE (Risk and Resilience Explored) report produced by the Vice Mental Health Guide (2015) revealed that 34% of gay, lesbian and bisexual youth (under 26 years of age) had attempted suicide at least once in their lifetime. In line with these findings, Nystedt et al. (2019) reported that bisexual and gay men, as well as bisexual women, showed a significantly higher likelihood of suicidal thoughts and significantly higher odds of suicide attempts compared with heterosexual men and women. Consistently, among women, those who died by suicide and belonged to sexual minority groups were 65% more likely to have disclosed suicidal thoughts and behaviors than heterosexual women (Clark & Blosnich, 2023).

One possible explanation for the elevated suicide risk among individuals with a sexual minority orientation is their exposure to a wide range of stressors, particularly those related to identity (Heisel et al., 2003; Hirsch et al., 2017; Keshoofy et al., 2023; I. Meyer, 2003; I. H. Meyer et al., 2021; Rosario et al., 2002). In this regard, the main factors identified as contributing to suicide risk include homophobic bullying, as well as difficulties within family and educational environments related to being gay, lesbian, or bisexual (Vice Mental Health Guide, 2015). Sexual minority individuals also experience higher rates of victimization and a greater risk of intimate partner violence than heterosexual individuals, in addition to facing social stigma and social exclusion (Arnoud et al., 2024; Baiocco et al., 2014; Centers for Disease Control and Prevention [CDC], 2024; Edwards et al., 2015; I. H. Meyer et al., 2021).

In this regard, the internalization of sexual stigma has become a significant variable in the analysis of behaviors related to suicide (Baiocco et al., 2014). This association may stem from diminished life satisfaction, precipitated by reduced relational intimacy and lower levels of social support in the face of homonegativity within salient social contexts (Baiocco et al., 2014; Exline et al., 2000; Pargament et al., 2000). Among these minority groups, religiosity has been linked to heightened internalization of sexual stigma (Barnes & Meyer, 2012; Lefevor et al., 2021; Wolf & Platt, 2022), potentially giving rise to conflicts between religiously transmitted messages and the sexual orientation with which individuals identify (Exline et al., 2000; Gibbs & Goldbach, 2015; Lefevor et al., 2021; Pargament et al., 2000).

In general terms, among the variables most closely associated with suicide attempts are hopelessness and depression, with hopelessness exhibiting greater explanatory relevance than depression. Hopelessness, as a clinical symptom of depression, is directly associated with behaviors aimed at ending perceived psychological distress (Beck et al., 1993).

Beck (1963) proposed that hopelessness constitutes one of the central characteristics leading to depression and suicide. Kazdin et al. (1983) argued that hopelessness may develop or be experienced over days or extended periods, shaping an individual’s relationship with the future. Hopelessness is characterized by a profound sense of demoralization, the inability to envision that something better may occur, or the loss of confidence in the possibility of change (Beck, 1963; Hirsch et al., 2017; Klonsky et al., 2012).

Data from the Youth Risk Behavior Survey conducted in Washington, DC (District of Columbia Public Schools, 2007) showed that 40% of young people who reported a sexual minority orientation indicated feeling sad or hopeless during the previous two weeks, compared with 26% of heterosexual youth (District of Columbia Public Schools, 2007). Numerous studies have corroborated the relevance of hopelessness as a risk factor for suicidal ideation, suicide attempts, and death by suicide (Abramson et al., 2000; Harwood et al., 2001; Kuo et al., 2005; Nock et al., 2010; Ribeiro et al., 2018; Ryan et al., 2024). In the meta-analysis conducted by Ribeiro et al. (2018), hopelessness was reported to have the strongest effect on suicidal ideation (OR = 2.19; 95% CI: 1.60–3.00); estimates were weaker when predicting suicide attempts (OR = 1.95; 95% CI: 1.59–2.39) and suicide death (OR = 1.98; 95% CI: 1.46–2.69). According to studies conducted in the Spanish population in 2013 by the Spanish State Federation of Lesbians, Gays, Transgender, and Bisexual People (FELGTB), hopelessness showed a high correlation with the risk of suicidal ideation. By contrast, other studies have reported substantially weaker associations (Holma et al., 2014; Ribeiro et al., 2012) or even non-significant effects (Nock & Banaji, 2007).

Similar evidence was reported in a study of 67,359 individuals that examined patterns of self-reported health according to sexual orientation and gender identity. Sexual minority individuals were more likely to report stress or worry; compared with heterosexual individuals, bisexual participants reported higher levels of hopelessness and suicidal ideation (Conron et al., 2010). In turn, Mozumder et al. (2023) reported that 32.4% of gay men had experienced a suicide attempt, and nearly half (47.1%) had a history of suicidal ideation, with 40.2% reporting self-harm behaviors. Compared with a heterosexual sample, gay men showed a higher prevalence of behaviors associated with hopelessness and suicide risk.

Another construct whose relationship with suicide risk and suicidal behaviors has been extensively examined is life satisfaction (Borzumato-Gainey et al., 2009; Mahmoud et al., 2012; O’Brien et al., 2023; Tedrus et al., 2023; Thatcher et al., 2002; Toukhy et al., 2024; Yildirim et al., 2023). Life satisfaction can be defined as an individual’s overall evaluation of their life (Diener & Biswas-Diener, 2002) and represents one of the core components of subjective well-being (Tedrus et al., 2023). It is a unidimensional construct that reflects how individuals evaluate their lives without imposing specific criteria for what constitutes a good life, leaving such judgments to the respondent (Diener et al., 1985). Life satisfaction is considered an aspect of mental health, with low levels being associated with mental health problems such as depressive symptoms and suicide (Borzumato-Gainey et al., 2009; Mahmoud et al., 2012; Rönkä et al., 2013; St. John et al., 2015; L. Wu, 2011).

Life satisfaction has been consistently linked to suicidal behaviors. In this regard, Thatcher et al. (2002) reported that low life satisfaction increases suicide risk. Accordingly, life satisfaction has been shown to be associated with greater severity of suicidal ideation through its relationship with depression (Chamizo-Nieto & Rey, 2023; Toukhy et al., 2024). As a risk factor, low life satisfaction is also associated with a higher number of negative life events (Luhmann et al., 2013). In addition, life dissatisfaction has also been considered a risk factor for non-suicidal self-injury (Adrian et al., 2011; Kress et al., 2015; Muehlenkamp & Brausch, 2012; Wang et al., 2025), although some findings contradict this association (Rotolone & Martin, 2012). Overall, high levels of life satisfaction function as a protective factor against depression and suicide (Dogra et al., 2011; O’Brien et al., 2023).

It has been observed that sexual orientation interacts with gender in predicting life satisfaction (Matías & Matud, 2023; Mohr & Fassinger, 2006). These studies identified an interaction between sexual orientation and gender, whereby lesbian or bisexual women reported higher levels of severe depressive symptoms and lower life satisfaction than heterosexual women. By contrast, life satisfaction scores among heterosexual individuals were very similar to those of gay or bisexual men. International research consistently indicates that gay, lesbian and bisexual individuals report lower life satisfaction compared with their heterosexual counterparts (Gómez et al., 2021; Morell-Mengual et al., 2017; Pachankis & Bränström, 2018; Powdthavee & Wooden, 2015).

A third construct that has also been examined in relation to suicide is spirituality (Alexandre Silva de Almeida et al., 2025; Danhauer et al., 2013; Jacob et al., 2019; Kleiman & Liu, 2014; Lawrence et al., 2016; O’Reilly & Rosato, 2015; Stack & Laubepin, 2019; A. Wu et al., 2015). Spirituality is related to the way in which, within a physically grounded environment, individuals experience the present moment and the interaction between the “self with oneself” and with the “other–environment”; that is, a form of verbal regulation that functions as a belief system attuned to environmental contingencies (Arbinaga et al., 2021). This conception of spirituality does not propose that spiritual experiences exist outside physical reality. Therefore, conceptualizing spirituality as a contextual behavioral construct is of relevance, since behaviors (operants) are under contextual control, which means they can be directly modified (Gentili et al., 2019).

Spirituality and religion-based coping strategies, which are generally considered adaptive (Park et al., 2018), may function as effective resources in populations facing health-related difficulties (Arbinaga et al., 2021; Jacob et al., 2019; Joaquín-Mingorance et al., 2019; Stack & Laubepin, 2019; Yang et al., 2016) and have been associated with higher quality of life (Finck et al., 2018), lower mood disturbance (Ringwald et al., 2016), and fewer depressive symptoms (Avis et al., 2013). Accordingly, spirituality is assumed to be associated with greater psychological well-being and may act as a protective factor in the face of adverse situations (Alexandre Silva de Almeida et al., 2025; Danhauer et al., 2013).

The constructs of religiosity and spirituality have traditionally been regarded as protective factors against suicide and as facilitators in the bereavement process following suicide (Alexandre Silva de Almeida et al., 2025; Jacob et al., 2019; Kleiman & Liu, 2014; Lawrence et al., 2016; O’Reilly & Rosato, 2015; Stack & Laubepin, 2019; A. Wu et al., 2015). However, some research has questioned this protective role (Exline et al., 2014; Gibbs & Goldbach, 2015; Lefevor et al., 2022b). Tensions between sexual identity and the religious beliefs, norms, or messages a person receives are associated with a higher risk of suicidal ideation (Exline et al., 2014; Gibbs & Goldbach, 2015). Sexual minority individuals face a set of unique risks related to both their sexual orientation and their religious or spiritual experiences. When suicidal ideation is compared between gay–lesbian and bisexual individuals who are actively engaged in religious practice and those who are not, similar levels of suicidal ideation have been reported; however, actively religious individuals show higher levels of religiosity and spirituality (Lefevor et al., 2022a).

Several variables have been identified as increasing the risk of suicidal ideation—such as positive religious coping, interpersonal religious struggles, internalized homonegativity, and concealment. In contrast, others have been found to reduce risk, including the resolution of conflicts between sexual and religious identities, family support, and peer support. Differential patterns across these factors have been highlighted between groups (Lefevor et al., 2022a; Stuhlsatz et al., 2021; Wolf & Platt, 2022).

The present study aimed to examine the influence of hopelessness, life satisfaction, and spirituality on suicide risk, considering these effects as a function of sexual orientation. The first hypothesis posited that participants with higher levels of hopelessness would exhibit a greater risk of suicide than those with lower levels, with a stronger effect expected among sexual minority groups. On the other hand, the second hypothesis proposed that life satisfaction would serve as a protective factor against suicide risk, such that individuals classified as being at high suicide risk will report significantly lower levels of life satisfaction; this pattern being particularly pronounced among sexual minority groups. Finally, the third hypothesis posited that higher scores in spirituality will be associated with a lower risk of suicide, regardless of sexual orientation.

## 2. Materials and Methods

### 2.1. Study Design

This research employed a cross-sectional observational design using online questionnaires.

### 2.2. Participants

A total of 553 individuals completed the instruments. Participants self-identified as heterosexual (n = 212, 38.3%), gay or lesbian (n = 182, 32.9%), bisexual (n = 138, 25.0%), transgender (n = 14, 2.5%), demisexual (n = 3, 0.5%), polysexual (n = 1, 0.2%), and pansexual (n = 3, 0.5%). Due to the low number of responses from individuals identifying as transgender, demisexual, polysexual, and pansexual, as well as the substantial differences among these groups and the resulting impossibility of forming a homogeneous fourth group, it was decided to exclude them from the study.

Consequently, a total of 532 individuals participated in the study (37.6% male), with ages ranging from 18 to 74 years. The age distribution was as follows: 18–33 years (65.4%), 34–49 years (23.9%), 50–65 years (10.2%), and 66–74 years (0.4%). The sample had a mean age of 31.15 years (*SD* = 12.002). Regarding educational level, 7.7% reported having completed basic education, 33.6% reported secondary education, and 58.6% reported having completed university studies. With respect to sexual orientation, 39.8% (*n* = 212) identified as heterosexual, 34.2% (*n* = 182) as gay–lesbian, and 25.9% (*n* = 138) as bisexual.

### 2.3. Instruments

An ad hoc interview was developed to collect sociodemographic information (year of birth, biological sex [male/female], and educational level [basic/secondary/university]). In addition, participants classified themselves according to the extent or type of religious belief as follows: (1) Non-Believer (NB)—I have no doubt; I do not believe in the existence of divine, superior, or transcendent beings beyond the realm of nature; (2) Non-Practicing Believer (NPB)—I believe in the existence of one or more divine or transcendent beings, but months may pass without attending religious services and/or religious events; and (3) Practicing Believer (PB)—I believe in the existence of one or more divine or transcendent beings and usually attend religious services and/or religious events, attempting to fulfill the precepts of my religion.

Suicide risk was assessed using the *Plutchik Suicide Risk Scale* (Plutchick et al., 1989) in its Spanish adaptation (Rubio et al., 1998). The scale consists of 15 dichotomous items (Yes/No). Each affirmative response is scored as one point, yielding a total score ranging from 0 to 15, with higher scores indicating greater suicide risk. The authors of the Spanish validation identified a score of six or higher as the cutoff point for high suicide risk. The Spanish-adapted version showed high internal consistency (Cronbach’s α = 0.90), with sensitivity and specificity of 88% at a cutoff score >6. In the present study, the scale demonstrated acceptable internal consistency (Cronbach’s α = 0.795).

Hopelessness was assessed using the *Hopelessness Scale* (Beck et al., 1974) in its Spanish translation and adaptation (Aguilar et al., 1995). The scale consists of 20 true/false items and is designed to measure negative expectations about the future. A cutoff score of 8 or higher indicates a high level of hopelessness. The original version showed high internal consistency (KR-20 = 0.93). In the present study, internal consistency was assessed using Cronbach’s alpha (α = 0.839).

Life satisfaction was assessed using the *Satisfaction with Life Scale* (Diener et al., 1985) in its Spanish translation and adaptation (Atienza et al., 2003; Pons et al., 2000). The scale consists of five items. Participants are asked to indicate their level of agreement with each statement on a 7-point Likert scale (ranging from 1 = strongly disagree to 7 = strongly agree). Total scores range from 5 to 35, with higher scores indicating greater life satisfaction. The original scale demonstrated good internal consistency (Cronbach’s α = 0.87). In the present study, the scale showed high internal consistency (Cronbach’s α = 0.883).

Spirituality was assessed using the *Beliefs and Values Scale* (King et al., 2006) in its Spanish translation (Carrasco, 2015). The scale consists of 20 items rated on a 5-point Likert scale ranging from 0 (*strongly disagree*) to 4 (*strongly agree*). The original version demonstrated high internal consistency (α = 0.93). In the present study, the scale showed excellent reliability (α = 0.952).

### 2.4. Procedure

Data were collected by contacting two Spanish LGTBI organizations (Fundación Triángulo and Federación Arcoíris de Andalucía), which disseminated the link to the online questionnaire through their social media channels. In addition, information about the study and the link to the online questionnaires were shared in several LGTBI online forums to invite participation. Finally, the study was disseminated among students enrolled in various courses at the University of Huelva. Participation was voluntary and anonymous, and participants did not receive any compensation for taking part, spending about seven or eight minutes completing the survey. All participants were required to be at least 18 years old and to read, sign, and provide informed consent; otherwise, they were not able to complete the questionnaires.

### 2.5. Statistical Analyses

Descriptive analyses (frequencies, percentages, means, and standard deviations) were conducted to characterize the main study variables. Group comparisons of quantitative variables were performed using independent-samples *t* tests. Effect sizes were estimated using Cohen’s *d* (<0.20 = small, 0.20–0.80 = medium, >0.80 = large). The internal consistency of the instruments was assessed using Cronbach’s alpha (α). Comparisons between categorical variables were conducted using chi-square (χ^2^) tests, with effect sizes estimated using Phi (0.10–0.29 = small, 0.30–0.49 = moderate, ≥0.50 = large) or Cramér’s V (<0.20 = small, 0.20–0.60 = moderate, >0.60 = large), as appropriate. For quantitative variables with more than two categories, analyses of variance (ANOVAs) were conducted using Snedecor’s F statistic, followed by Bonferroni post hoc tests. Effect sizes for ANOVA were calculated using eta squared (η^2^), with values of 0.01–0.05 indicating small, 0.06–0.13 medium, and ≥0.14 large effects. Stepwise linear regression analysis was employed and all statistical assumptions necessary for the analyses were examined (e.g., homoscedasticity, multicollinearity assessed via variance inflation factor [VIF]). Associations among variables were analyzed using Pearson correlations, and hierarchical linear regression was used to identify predictors of suicide risk. No missing data were observed, as completion of all items was required to proceed with the questionnaires. All analyses were performed using SPSS (IBM SPSS Statistics, version 25.0; IBM Corp., Armonk, NY, USA).

## 3. Results

Table 1 presents the basic sociodemographic characteristics of the sample. As shown, the groups differed significantly in age, with a medium effect size (η^2^ = 0.07). Participants who identified as bisexual were younger than both heterosexual participants (*p* < 0.001) and gay–lesbian participants (*p* < 0.001). No significant age differences were observed between the heterosexual and gay–lesbian groups.

In addition, significant differences were found as a function of biological sex, with a moderate effect size (Cramér’s *V* = 0.233). Specifically, heterosexual participants were more likely to be women, whereas gay–lesbian participants were predominantly men. No significant sex differences were observed within the bisexual group.

Regarding reported educational level, significant differences were observed, with a small effect size (Cramér’s *V* = 0.105). Participants identifying as heterosexual predominantly reported having completed university education, whereas bisexual participants tended to report secondary education. No statistically significant differences were observed within the group identifying as gay–lesbian.

Finally, with respect to religious identification, significant differences were found across groups, with a moderate effect size (Cramér’s *V* = 0.221). In this regard, heterosexual participants were more likely to identify as believers, either practicing or non-practicing, whereas bisexual and gay–lesbian participants primarily reported not being believers.

With respect to hopelessness scores (Table 2), significant differences were observed, driven primarily by the group identifying as bisexual, which obtained higher scores than the other groups, although the effect size was small (η^2^ = 0.05). Post hoc comparisons among the three groups indicated that *a* = *b* (*p* = 0.397), *a* < *c* (*p* < 0.001), and *b* < *c* (*p* = 0.001).

Similarly, the three groups differed significantly in life satisfaction scores, also with a small effect size (η^2^ = 0.05). Sexual minority groups—particularly bisexual participants—reported lower levels of life satisfaction. Post hoc comparisons indicated that *a* > *b* (*p* = 0.025), *a* > *c* (*p* < 0.001), and *b* > *c* (*p* = 0.043).

With respect to spirituality scores, the heterosexual group emerged as the most spiritual, although the effect size was small (η^2^ = 0.05). No differences were observed between the gay–lesbian and bisexual groups, as indicated by the post hoc comparisons [*a* > *b* (*p* < 0.001), *a* > *c* (*p* < 0.001), and *b* = *c* (*p* = 1.00)].

From the perspective of religious identification, significant differences in spirituality scores were observed among Non-Believers (NB) (*M* = 40.17, *SD* = 11.96), Non-Practicing Believers (NPB) (*M* = 65.34, *SD* = 12.08), and Practicing Believers (PB) (*M* = 76.82, *SD* = 11.26), *F*(2,531) = 349.72, *p* < 0.001, with a large effect size (η^2^ = 0.57). Post hoc Bonferroni comparisons indicated that NB < NPB (*p* < 0.001), NB < PB (*p* < 0.001), and NPB < PB (*p* < 0.001).

In contrast, no significant differences were found according to reported religiosity (NB, NPB, PB) in suicide risk scores, *F*(2,531) = 0.74, *p* = 0.479, or in life satisfaction, *F*(2,531) = 0.50, *p* = 0.605. A significant effect was observed for hopelessness, *F*(2,531) = 3.29, *p* = 0.038; however, Bonferroni post hoc comparisons revealed no statistically significant differences between the three groups.

Finally, the three groups differed significantly in suicide risk scores, with a medium effect size (η^2^ = 0.13). Post hoc comparisons revealed that *a* < *b* (*p* < 0.001), *a* < *c* (*p* < 0.001), and *b* < *c* (*p* = 0.010).

Accordingly, when participants were classified by sexual orientation and suicide risk scores were dichotomized into high suicide risk versus low suicide risk, the results (Table 3) indicated that 41.2% of gay–lesbian participants and 52.9% of bisexual participants were classified as being at high suicide risk, compared with 15.6% of heterosexual participants. This association showed a moderate effect size (Cramér’s *V* = 0.331).

The data indicate that of the individuals classified in the high suicide risk category, 18.2% identified as heterosexual, 41.4% as gay or lesbian, and 40.3% as bisexual. In contrast, among those categorized in the low suicide risk group, 51.0% identified as heterosexual, 30.5% as gay or lesbian, and 18.5% as bisexual.

When examining the behavior of the study variables as a function of dichotomized suicide risk (Table 4), individuals at high suicide risk showed significantly higher levels of hopelessness than those at low risk, with a large effect size (Cohen’s *d* = 1.183), and significantly lower levels of life satisfaction, also with a large effect size (Cohen’s *d* = 0.92). However, no significant differences were observed between the groups in spirituality scores.

In this regard, individuals classified in the high hopelessness category (*n* = 106) showed significantly higher suicide risk scores (*M* = 7.92, *SD* = 2.85) than those classified as having low hopelessness (*n* = 426; *M* = 3.64, *SD*= 2.64), *t* = 14.69, *p* < 0.001, with a large effect size (Cohen’s *d* = 1.56).

When examining the distribution of sexual orientation across hopelessness categories (high vs. low), significant differences were observed, χ^2^(4,532) = 17.92, *p* < 0.001, although the effect size was small (Cramér’s *V* = 0.184). The groups driving these differences were heterosexual participants, who tended to fall within the low hopelessness category (43% of the group, *n* = 183), and bisexual participants, who were significantly overrepresented in the high hopelessness category (41.5% of the group, *n* = 44). By contrast, participants identifying as gay or lesbian did not show statistically significant differences across hopelessness categories.

Table 5 presents the Pearson correlation coefficients among the study variables. Suicide risk scores were positively and significantly correlated with hopelessness and negatively correlated with life satisfaction. In contrast, suicide risk showed no significant association with spirituality scores.

Additionally, hopelessness was negatively and significantly correlated with both spirituality and life satisfaction. Life satisfaction, however, did not show a significant association with spirituality.

After verifying the assumptions of the regression models—including homoscedasticity and multicollinearity assessed via the variance inflation factor (VIF)—significant models were identified. In the final model, VIF values indicated acceptable levels of collinearity for hopelessness (VIF = 1.606), life satisfaction (VIF = 1.573), and spirituality (VIF = 1.027).

When a linear regression analysis was conducted on the total sample, with suicide risk as the outcome variable and hopelessness, life satisfaction, and spirituality as predictors (Table 6), the resulting model was significant and accounted for 36.2% of the variance. Hopelessness emerged as a strong predictor (β = 0.448), whereas life satisfaction acted as a protective factor, showing a negative predictive contribution of β = −0.218. Spirituality did not show a significant predictive effect.

When regression analyses were conducted separately for each sexual orientation group (Table 7), differential patterns emerged. All three groups yielded significant models; notably, the bisexual group showed the greatest explanatory power for suicide risk, accounting for 42.8% of the variance, followed by the gay–lesbian group (34.2%) and the heterosexual group (29.9%).

Although hopelessness predicted a very similar proportion of across the three groups (β = 0.446 in heterosexuals, β = 0.447 in gay–lesbian, and β = 0.457 in bisexuals), life satisfaction showed greater predictive strength in the bisexual group (β = −0.241). In all groups, the predictive effect of life satisfaction was negative, indicating a protective role, although its statistical significance in the heterosexual group was marginal.

By contrast, spirituality did not reach statistical significance in the heterosexual group, although its negative coefficient (β = −0.64) suggested a modest protective tendency. However, in the gay–lesbian (β = 0.133) and bisexual (β = 0.214) groups, spirituality did not exhibit a protective effect; this non-protective association was particularly pronounced in the bisexual group.

## 4. Discussion

The present study aimed to examine the influence of hopelessness, life satisfaction, and spirituality on suicide risk, considering these effects as a function of sexual orientation.

The first hypothesis posited that participants with higher levels of hopelessness would exhibit a greater risk of suicide than those with lower levels, with a stronger effect expected among sexual minority groups. The findings partially supported this hypothesis. Individuals with high hopelessness scores showed a markedly higher suicide risk, with a large effect size. However, when sexual orientation was taken into account, hopelessness demonstrated a similar predictive capacity for suicide risk across all three groups.

On the other hand, participants identifying as bisexual reported higher levels of hopelessness than the other two groups, which did not differ significantly from each other. In this regard, heterosexual participants were predominantly classified within the low hopelessness group, whereas bisexual participants were overrepresented in the high hopelessness group. Participants identifying as gay or lesbian showed a more even distribution across both hopelessness categories.

Overall, hopelessness demonstrated substantial predictive power for suicide risk (β = 0.448), supporting previous research that has identified hopelessness as a central characteristic in suicide-related processes (Abramson et al., 2000; Beck, 1963; Harwood et al., 2001; Hirsch et al., 2017; Kazdin et al., 1983; Klonsky et al., 2012; Kuo et al., 2005; Nock et al., 2010; Ribeiro et al., 2018; Ryan et al., 2024).

Indeed, several meta-analyses have reported that hopelessness shows the strongest effect on suicidal ideation, with smaller but still significant effects on suicide attempts and death by suicide (Ribeiro et al., 2018; Riera-Serra et al., 2024). However, these findings are not fully consistent with those reported by Holma et al. (2014), who found weak associations between hopelessness and suicidal ideation, or by Nock and Banaji (2007), who detected no significant relationships.

When sexual orientation is examined differentially, the present findings partially support previous evidence indicating that sexual minority individuals, compared with those identifying as heterosexual, are more likely to report high levels of hopelessness (District of Columbia Public Schools, 2007; Federación Estatal de Lesbianas, Gais, Transexuales y Bisexuales, 2013), particularly individuals identifying as bisexual (Conron et al., 2010). However, no differences in hopelessness scores were observed between gay–lesbian and heterosexual participants, contrary to the findings reported by Mozumder et al. (2023), who identified higher hopelessness levels among gay and lesbian individuals. Notably, hopelessness showed a similar predictive capacity for suicide risk across all three groups, regardless of sexual orientation.

With respect to the second hypothesis, the results fully supported its prediction. Life satisfaction was confirmed to act as a protective variable against suicide risk. Specifically, individuals at high suicide risk reported lower levels of life satisfaction. These findings are consistent with previous research linking low life satisfaction to suicide (Borzumato-Gainey et al., 2009; Mahmoud et al., 2012; Rönkä et al., 2013; St. John et al., 2015; Thatcher et al., 2002; L. Wu, 2011), often through its association with depression (Chamizo-Nieto & Rey, 2023; Toukhy et al., 2024).

When considering the total sample, life satisfaction showed a significant negative predictive effect (β = −0.218) on suicide risk. Thus, its protective role against suicide is reaffirmed, in line with previous findings reported by Dogra et al. (2011) and O’Brien et al. (2023).

When life satisfaction was analyzed as a function of sexual orientation, differential patterns emerged across the three groups. Heterosexual participants reported the highest levels of life satisfaction, followed by gay and lesbian participants, with bisexual participants reporting the lowest levels. However, a stronger protective effect of life satisfaction in predicting suicide risk was observed among sexual minority groups, particularly among bisexual participants. These findings are consistent with studies showing that sexual orientation predicts levels of life satisfaction (Matías & Matud, 2023; Mohr & Fassinger, 2006). Likewise, a substantial body of international research has consistently reported lower life satisfaction among gay, lesbian and bisexual individuals compared with their heterosexual counterparts (Gómez et al., 2021; Morell-Mengual et al., 2017; Pachankis & Bränström, 2018; Powdthavee & Wooden, 2015).

A useful framework for understanding the differences observed in life satisfaction as a function of sexual orientation is Minority Stress Theory (MST), proposed by Brooks (1981) and further developed by I. Meyer (2003) and I. H. Meyer et al. (2021). Previous research has shown that low life satisfaction increases suicide risk by being associated with a greater number of negative life events (Luhmann et al., 2013), a pattern that is particularly evident among sexual minority populations (Heisel et al., 2003; Hirsch et al., 2017; Keshoofy et al., 2023; I. Meyer, 2003; I. H. Meyer et al., 2021). Within this framework, a key factor that promotes life satisfaction among sexual minorities—by facilitating coping with adverse events—is the degree of identity integration (Rosario et al., 2011), which Bregman et al. (2013) identify as an important outcome of acceptance within the family context.

Along with the Minority Stress Theory, widely used to explain the higher prevalence of health problems among sexual minorities, heterosexism should also be considered as a set of practices aligned with this framework (Chang et al., 2020; Pharr & Batra, 2024; Trujillo et al., 2020). Heterosexism, understood as a system of beliefs and practices that privileges heterosexuality and discriminates against other sexual orientations, increases suicide risk among sexual minorities through mechanisms such as chronic stigma and discrimination, reduced social support, heightened minority stress, impaired emotion regulation, and the internalization of social rejection, among others (Chang et al., 2020; Pharr & Batra, 2024; Trujillo et al., 2020).

Finally, concerning the third hypothesis and considering the sample as a whole, the results do not support the assumption that individuals with higher levels of spirituality report a lower risk of suicide. Accordingly, spirituality cannot be considered a protective variable against suicide risk in the overall sample. In line with this, no significant differences in spirituality were observed when comparing individuals with high versus low suicide risk. Additionally, heterosexual participants reported higher levels of spirituality than gay–lesbian and bisexual participants, who did not differ from each other. However, when each group was analyzed separately, spirituality showed a significant and positive predictive association with suicide risk in the gay–lesbian and bisexual groups, indicating that, contrary to expectations, spirituality did not function as a protective factor within these sexual minority groups.

These findings do not support previous research suggesting that spirituality and religiosity may reduce the impact of adverse situations (Alexandre Silva de Almeida et al., 2025; Danhauer et al., 2013) and are associated with lower mood disturbance and suicidal ideation (Avis et al., 2013; Ringwald et al., 2016).

Conversely, the findings align with a growing body of scholarship that challenges the traditional assumption that spirituality and religiosity function as protective factors against suicide risk (Baiocco et al., 2014; Barnes & Meyer, 2012; Exline et al., 2000; Gibbs & Goldbach, 2015; Lefevor et al., 2021; Wolf & Platt, 2022). Empirical evidence indicates that, among sexual minorities, spirituality may be associated with heightened psychological distress, increased suicidal ideation, and poorer emotional adjustment, particularly when it is embedded within religious environments that sustain negative discourses regarding sexual diversity (Barnes & Meyer, 2012; Lefevor et al., 2021; Wolf & Platt, 2022). Within such contexts, tensions between sexual identity and religious belief emerge, generating a significant psychosocial stressor that can exacerbate vulnerability to suicidal behavior (Exline et al., 2000; Gibbs & Goldbach, 2015; Lefevor et al., 2021).

In this framework, spirituality ceases to operate as a positive coping resource and instead becomes a source of spiritual strain, a phenomenon conceptualized in the literature as negative religious coping (Baiocco et al., 2014; Exline et al., 2000; Gibbs & Goldbach, 2015; Lefevor et al., 2021). This form of coping—characterized by punitive interpretations of faith, perceptions of divine abandonment, or beliefs in spiritual punishment—has been consistently associated with elevated levels of depression, hopelessness, and suicidal ideation (Exline et al., 2014; Pargament et al., 2000).

When suicide risk scores were compared according to self-reported religiosity (non-believers, non-practicing believers, and practicing believers), no significant differences were observed between groups. However, significant differences in spirituality were found across the three religiosity categories. These results are consistent with studies showing that, when comparing suicidal ideation among gay–lesbian and bisexual individuals who are actively engaged in religious practice versus those who are not, similar levels of suicidal ideation are observed, despite higher levels of religiosity and spirituality among actively religious individuals (Lefevor et al., 2022a; Stuhlsatz et al., 2021; Wolf & Platt, 2022).

Although the present study contributes to the understanding of suicide risk among sexual minority populations, several limitations should be considered when interpreting the findings and addressed in future research. These include the inherent limitations associated with the use of self-report questionnaires and online data collection. In addition, the study’s cross-sectional design precludes establishing causal relationships between variables. On the other hand, the use of a non-probabilistic sample that is not representative of the various groups may hinder the generalization of the results obtained. In this regard, the low or nonexistent participation of specific groups (transgender, demisexual, etc.) becomes a limiting factor when interpreting the findings. Likewise, when considering biological sex in some orientations, the resulting group size was small; therefore, this represents a limitation in terms of control and proportionality.

Furthermore, future studies should conduct more fine-grained analyses that jointly consider gender and sexual orientation in relation to the variables examined. It would also be valuable to investigate how different forms of homophobia (personal, institutional, and internalized), as well as variations in victimization—such as the greater vulnerability of bisexual individuals to domestic violence, hate crimes, and the lack of justice in society—are associated with the three variables analyzed (hopelessness, life satisfaction, and spirituality) and their implications for suicidal behaviors. Examining these structural and interpersonal stressors alongside psychological ones would contribute to a more nuanced understanding of the mechanisms shaping suicide risk among sexual minority populations. Finally, the inclusion of clinical variables (e.g., depression) and contextual factors (e.g., socio-familial variables) related to sexual orientation would allow for a more comprehensive understanding of their role in suicide risk within sexual minority populations.

## 5. Conclusions

In conclusion, 52.9% of bisexual participants and 41.2% of gay and lesbian participants were classified as being at high suicide risk, compared with 15.6% of heterosexual participants. Bisexual individuals reported the highest levels of hopelessness. Hopelessness demonstrated a strong and significant predictive capacity for suicide risk, regardless of sexual orientation. Conversely, life satisfaction acted as a protective factor against suicide risk, particularly among sexual minority groups. Finally, spirituality showed a positive and significant predictive association with suicide risk among sexual minority participants, whereas no significant association was observed among heterosexual participants.

## Figures and Tables

**Table 1:** Sample characteristics according to sexual orientation.

	Total 532	Heterosexual (a) 212 (39.8)	Gay–Lesbian (b) 182 (34.2)	Bisexual (c) 138 (25.9)		*p*
Age	31.15 (12.002)	32.82 (12.618)	33.46 (12.334)	25.55 (8.319)	*F*_(2,531)_ = 22.037	<0.001
Biological Sex					*χ*^2^_(2,532)_ = 28.915	<0.001
Male	200 (37.6)	69 (32.5)	96 (52.7)	35 (25.4)		
Female	332 (62.4)	143 (67.5)	86 (47.3)	103 (74.6)		
Educational Level					*χ*^2^_(4,532)_ = 11.734	0.019
Basic	41 (7.7)	16 (7.5)	14 (7.7)	11 (8.0)		
Secondary	179 (33.6)	60 (28.3)	57 (31.3)	62 (44.9)		
University	312 (58.6)	136 (64.2)	111 (61.0)	65 (47.1)		
Religious Beliefs					*χ*^2^_(4,532)_ = 52.068	<0.001
NB	270 (50.8)	69 (32.5)	115 (63.2)	86 (62.3)		
NPB	224 (42.1)	116 (54.7)	62 (34.1)	46 (33.3)		
PB	38 (7.1)	27 (12.7)	5 (2.7)	6 (4.3)		

Note: For quantitative variables, M (SD), for categorical variables, n (%); NB: non-believer, NPB non-practicing believer, PB: practicing believer.

**Table 2:** Scores on the variables -spirituality, hopelessness, life satisfaction, and suicide risk-according to sexual orientation.

	Total 532	Heterosexual (a) 212 (39.8)	Gay–Lesbian (b) 182 (34.2)	Bisexual (c) 138 (25.9)	*F*_(2,531)_	*p*
Suicide risk	4.50 (3.182)	3.17 (2.628)	4.95 (3.168)	5.94 (3.196)	39.714	<0.001
Hopelessness	4.72 (3.946)	3.97 (3.353)	4.55 (3.976)	6.09 (4.401)	12.846	<0.001
Life satisfaction	16.67 (4.631)	17.73 (4.193)	16.51 (4.874)	15.25 (4.568)	12.608	<0.001
Spirituality	53.39 (18.193)	58.45 (18.785)	50.63 (16.826)	49.24 (17.269)	14.613	<0.001

Note: For quantitative variables, M (SD), for categorical variables, n (%).

**Table 3:** Suicide risk (high vs. low) based on sexual orientation.

	Total 532	Heterosexual (a) 212 (39.8)	Gay–Lesbian (b) 182 (34.2)	Bisexual (c) 138 (25.9)	*χ* ^2^_(2,532)_	*p*
Risk of suicide					58.263	<0.001
High	181 (34.0)	33 (15.6)	75 (41.2)	73 (52.9)		
Low	351 (66.0)	179 (84.4)	107 (58.8)	65 (47.1)		

Note: For quantitative variables, M (SD), for categorical variables, n (%).

**Table 4:** Scores for the variables spirituality, hopelessness, and life satisfaction according to suicide risk (high vs. low).

	Total 532	High Risk of Suicide 181 (34.0)	Low Risk of Suicide 351 (66.0)	*t*	*p*
Hopelessness	4.72 (3.946)	7.46 (4.684)	3.30 (2.541)	11.117	<0.001
Life satisfaction	16.67 (4.631)	14.03 (4.716)	18.03 (3.955)	9.763	<0.001
Spirituality	53.39 (18.193)	51.82 (18.028)	54.19 (18.251)	1.424	0.155

Note: For quantitative variables, M (SD), for categorical variables, n (%).

**Table 5:** Pearson correlation coefficient between the variables studied.

		1	2	3	4
1	Suicide risk	1			
2	Hopelessness	0.575/<0.001	1		
3	Life satisfaction	−0.486/<0.001	−0.603/<0.001	1	
4	Spirituality	−0.064/0.143	−0.160/<0.001	0.072/0.099	1

Note: Pearson correlation coefficient/significance.

**Table 6:** Linear regression analysis, taking suicide risk as the predicted variable and spirituality, hopelessness, and life satisfaction as predictor variables, according to total sample.

Total Sample	*β*	*t*	*p*	*R* ^2^	*p*	*F*_(3,531)_	*p*
**Model 1**				0.362	<0.001	99.860	<0.001
Hopelessness	0.448	10.161	<0.001				
Life satisfaction	−0.218	4.999	<0.001				
Spirituality	0.024	0.676	0.499				

**Table 7:** Linear regression analysis, taking suicide risk as the predicted variable and spirituality, hopelessness, and life satisfaction as predictor variables, according to sexual orientation.

Heterosexual	*β*	*t*	*p*	*R* ^2^	*p*	*F*_(3,211)_	*p*
**Model 1**				0.299	<0.001	29.633	<0.001
Hopelessness	0.446	6.375	<0.001				
Life satisfaction	−0.137	1.983	0.049				
Spirituality	−0.064	1.074	0.284				
**Gay-Lesbian**	*β*	*t*	*p*	*R* ^2^	*p*	*F*_(3,181)_	*p*
**Model 1**				0.342	<0.001	30.859	<0.001
Hopelessness	0.447	5.986	<0.001				
Life satisfaction	−0.186	2.486	0.014				
Spirituality	0.133	2.164	0.032				
**Bisexual**	*β*	*t*	*p*	*R* ^2^	*p*	*F*_(3,137)_	*p*
**Model 1**				0.428	<0.001	33.374	<0.001
Hopelessness	0.457	5.222	<0.001				
Life satisfaction	−0.241	2.766	0.006				
Spirituality	0.214	3.239	0.002				

## Data Availability

This study adheres to the Transparency and Openness Promotion (TOP) guidelines and adheres to the Journal Article Reporting Standards (JARS), including transparent reporting of sample size determination, data exclusions, manipulations, and measures. All anonymized raw participant data are available at the Open Science Framework: https://doi.org/10.17605/OSF.IO/PTFBD.

## Abbreviations

The following abbreviations are used in this manuscript:

LGTBI	Lesbian, Gay, Transgender, Bisexual, Intersex
FELGTB	Federación Estatal de Lesbianas, Gais, Transexuales y Bisexuales
VIF	Variance Inflation Factor
NB	Non-Believer
NPB	Non-Practicing-Believer
PB	Practicing-Believer
MST	Minority Stress Theory

## References

[B1-behavsci-16-00406] Abdulai T. (2020). Trends of online news media reported suicides in Ghana (1997–2019). BMC Public Health.

[B2-behavsci-16-00406] Abramson L. Y., Alloy L. B., Hogan M. E., Whitehouse W. G., Gibb B. E., Hankin B. L., Cornette M. M., Joiner T., Rudd M. D. (2000). The hopelessness theory of suicidality. Suicide science.

[B3-behavsci-16-00406] Adrian M., Zeman J., Erdley C., Lisa L., Sim L. (2011). Emotional dysregulation and interpersonal difficulties as risk factors for nonsuicidal self-injury in adolescent girls. Journal of Abnormal Child Psychology.

[B4-behavsci-16-00406] Aguilar E., Hidalgo M., Cano R., López J., Campillo M., Hernández J. (1995). Estudio prospectivo de la desesperanza en pacientes psicóticos de inicio: Características psicométricas de la escala de desesperanza de beck en este grupo. Anales de Psiquiatría.

[B5-behavsci-16-00406] Alexandre Silva de Almeida V., Ossamu Shintani A., Reis-Vargas P., Cavalcante-Passos I., Moreira-Almeida A. (2025). Religiosity and spirituality in coping with suicide grief: A review. International Journal of Social Psychiatry.

[B6-behavsci-16-00406] Arbinaga F., Mendoza-Sierra M. I., Bohórquez M. R., Verjano-Cuellar M. I., Torres-Rosado L., Romero-Pérez N. (2021). Spirituality, religiosity and coping strategies among Spanish people diagnosed with cancer. Journal of Religion and Health.

[B7-behavsci-16-00406] Arnoud T. C. J., Linhares I. Z., Dos Reis Rodrigues G., Habigzang L. F. (2024). Dating violence victimization among sexual and gender diver-se adolescents in Brazil. Current Psychology.

[B8-behavsci-16-00406] Atienza F. L., Balaguer I., García-Merita M. L. (2003). Satisfaction with Life Scale: Analysis of factorial invariance across sexes. Personality and Individual Differences.

[B9-behavsci-16-00406] Avendaño-Prieto B. L., Betancort Montesinos M., Bernal-Aguirre A., González-Martínez L. A., Gómez-Sánchez S. M., Villalobos-Sánchez C. F. (2019). Celos, desesperanza e ideación suicida en población con orientación sexual diversa. Universitas Psychologica.

[B10-behavsci-16-00406] Avis N. E., Levine B., Naughton M. J., Case L. D., Naftalis E. (2013). Age-related longitudinal changes in depressive symptoms following breast cancer diagnosis and treatment. Breast Cancer Research Treatment.

[B11-behavsci-16-00406] Baca-Garcia E., Perez-Rodriguez M. M., Oquendo M. A., Keyes K. M., Hasin D. S., Grant B. F., Blanco C. (2011). Estimating risk for suicide attempt: Are we asking the right questions? Passive suicidal ideation as a marker for suicidal behavior. Journal of Affective Disorders.

[B12-behavsci-16-00406] Baiocco R., Ioverno S., Cerutti R., Santamaria F., Fontanesi L., Lingiardi V., Baumgartner E., Laghi F. (2014). Suicidal ideation in Spanish and Italian lesbian and gay young adults: The role of internalized sexual stigma. Psicothema.

[B13-behavsci-16-00406] Barnes D. M., Meyer I. H. (2012). Religious affiliation, internalized homophobia, and mental health in sexual minority individuals. American Journal of Orthopsychiatry.

[B14-behavsci-16-00406] Beck A. T. (1963). Thinking and depression: I. Idiosyncratic content and cognitive distortions. Archives of General Psychiatry.

[B15-behavsci-16-00406] Beck A. T., Steer R., Brown G. (1993). Dysfunctional attitudes and suicidal ideation in psychiatric outpatients. Suicide Life-Threatening Behavior.

[B16-behavsci-16-00406] Beck A. T., Weissman A., Lester D., Trexler L. (1974). The measurement of pessimism: The hopelessness Scale. Journal of Consulting and Clinical Psychology.

[B17-behavsci-16-00406] Blosnich J. R. (2022). Interpersonal and self-directed violence among sexual and gender minority populations: Moving research from prevalence to prevention. Current Epidemiology Reports.

[B18-behavsci-16-00406] Borzumato-Gainey C., Kennedy A., McCabe B., Degges-White S. (2009). Life satisfaction, self-esteem, and subjective age in women across the life span. Adultspan Journal.

[B19-behavsci-16-00406] Bregman H., Malik N., Page M., Makynen E., Lindahl K. (2013). Identity profiles in lesbian, gay, and bisexual youth: The role of family influences. Journal of Youth and Adolescence.

[B20-behavsci-16-00406] Brooks V. R. (1981). Minority stress and lesbian women.

[B21-behavsci-16-00406] Carrasco Y. (2015). Religion and its influence on health behaviors *[Unpublished Doctoral thesis, University of Huelva]*.

[B22-behavsci-16-00406] Centers for Disease Control and Prevention [CDC] (2024). Youth risk behavior survey data summary & trends report: 2013–2023.

[B23-behavsci-16-00406] Chamizo-Nieto M. T., Rey L. (2023). Cybervictimization and suicidal ideation in adolescents: A prospective view through gratitude and life satisfaction. Journal of Health Psychology.

[B24-behavsci-16-00406] Chang C. J., Fehling K. B., Selby E. A. (2020). Sexual minority status and psychological risk for suicide attempt: A serial multiple mediation model of social support and emotion regulation. Frontiers in Psychiatry.

[B25-behavsci-16-00406] Clark K. A., Blosnich J. R. (2023). Sexual orientation and disclosure of suicidal thoughts before suicide mortality. American Journal of Preventive Medicine.

[B26-behavsci-16-00406] Cochran S. D., Mays V. M. (2015). Mortality risks among persons reporting same-sex sexual partners: Evidence from the 2008 general social survey-national death index data set. American Journal of Public Health.

[B27-behavsci-16-00406] Conron K. J., Mimiaga M. J., Landers S. J. (2010). A population-based study of sexual orientation identity and gender differences in adult health. American Journal of Public Health.

[B28-behavsci-16-00406] Danhauer S. C., Douglas-Case L., Tedeschi R., Russell G., Vishnevsky T., Triplett K., Ip E. H., Avis N. E. (2013). Predictors of posttraumatic growth in women with breast cancer. Psycho-Oncology.

[B29-behavsci-16-00406] Diener E., Biswas-Diener R. (2002). Will money increase subjective well-being?. Social Indicators Research.

[B30-behavsci-16-00406] Diener E., Emmons R., Larsen R. J., Griffin S. (1985). The satisfaction with life scale. Journal of Personality Assessment.

[B31-behavsci-16-00406] District of Columbia Public Schools (2007). Youth risk behavior survey sexual minority baseline fact sheet: Senior high school YRBS 2007 baseline findings for GLBQ items.

[B32-behavsci-16-00406] Dogra A. K., Basu S., Das S. (2011). Impact of meaning in life and reasons for living to hope and suicidal ideation: A study among college students. SIS Journal of Projective Psychology and Mental Health.

[B33-behavsci-16-00406] Edwards K. M., Sylaska K. M., Barry J. E., Moynihan M. M., Banyard V. L., Cohn E. S., Walsh W. A., Ward S. K. (2015). Physical dating violence, sexual violence, and unwanted pursuit victimization: A comparison of incidence rates among sexual minority and heterosexual college students. Journal of Interpersonal Violence.

[B34-behavsci-16-00406] Erlangsen A., Drefahl S., Haas A., Bjorkenstam C., Nordentoft M., Andersson G. (2020). Suicide among persons who entered same-sex and opposite-sex marriage in Denmark and Sweden, 1989–2016: A binational, register-based cohort study. Journal of Epidemiology and Community Health.

[B35-behavsci-16-00406] Exline J. J., Pargament K. I., Grubbs J. B., Yali A. M. (2014). The religious and spiritual struggles scale: Development and initial validation. Psychology of Religion and Spirituality.

[B36-behavsci-16-00406] Exline J. J., Yali A. M., Sanderson W. C. (2000). Guilt, discord, and alienation: The role of religious strain in depression and suicidality. Journal of Clinical Psychology.

[B37-behavsci-16-00406] Federación Estatal de Lesbianas, Gais, Transexuales y Bisexuales (2013). Acoso escolar (y riesgo de suicidio) por orientación sexual e identidad de género: Fracaso del sistema educativo.

[B38-behavsci-16-00406] Finck C., Barradas S., Zenger M., Hinz A. (2018). Quality of life in breast cancer patients: Associations with optimism and social support. International Journal of Clinical and Health Psychology.

[B39-behavsci-16-00406] Gentili C., Rickardsson J., Zetterqvist V., Simons L. E., Lekander M., Wicksell R. K. (2019). Psychological flexibility as a resilience factor in individuals with chronic pain. Frontiers in Psychology.

[B40-behavsci-16-00406] Gibbs J. J., Goldbach J. (2015). Religious conflict, sexual identity, and suicidal behaviors among LGBT young adults. Archives of Suicide Research.

[B41-behavsci-16-00406] Glenn C. R., Kleiman E. M., Kellerman J., Pollak O., Cha C. B., Esposito E. C., Porter A. C., Wyman P. A., Boatman A. E. (2020). Annual research review: A meta-analytic review of worldwide suicide rates in adolescents. Journal of Child Psychology and Psychiatry.

[B42-behavsci-16-00406] Gómez F., Cumsille P., Barrientos J. (2021). Mental health and life satisfaction on Chilean gay men and lesbian women: The role of perceived sexual stigma, internalized homophobia, and community connectedness. Journal of Homosexuality.

[B43-behavsci-16-00406] Harwood D., Hawton K., Hope T., Jacoby R. (2001). Psychiatric disorder and personality factors associated with suicide in older people: A descriptive and case-contro study. International Journal of Geriatric Psychiatry.

[B44-behavsci-16-00406] Heisel M. J., Flett G. L., Hewitt P. L. (2003). Social hopelessness and college student suicide ideation. Archives of Suicide Research.

[B45-behavsci-16-00406] Hirsch J. K., Cohn T. J., Rowe C. A., Rimmer S. E. (2017). Minority sexual orientation, gender identity status and suicidal behavior: Serial indirect effects of hope, hopelessness and depressive symptoms. International Journal of Mental Health and Addiction.

[B46-behavsci-16-00406] Holma K. M., Haukka J., Suominen K., Valtonen H. M., Mantere O., Melartin T. K., Sokero T. P., Oquendo M. A., Isometsä E. T. (2014). Differences in incidence of suicide attempts between bipolar I and II disorders and major depressive disorder. Bipolar Disorder.

[B47-behavsci-16-00406] Hottes T. S., Bogaert L., Rhodes A. E., Brennan D. J., Gesink D. (2016). Lifetime prevalence of suicide attempts among sexual minority adults by study sampling strategies: A systematic review and meta-analysis. American Journal of Public Health.

[B48-behavsci-16-00406] Jacob L., Haro J. M., Koyanagi A. (2019). The association of religiosity with suicidal ideation and suicide attempts in the United Kingdom. Acta Psychiatrica Scandinavica.

[B49-behavsci-16-00406] Joaquín-Mingorance M., Arbinaga F., Carmona-Márquez J., Bayo-Calero J. (2019). Coping strategies and self-esteem in women with breast cancer. Annals of Psychology.

[B50-behavsci-16-00406] Kazdin A. E., French N. H., Unis A. S., Esveldt-Dawson K., Sherick R. B. (1983). Hopelessness, depression, and suicidal intent among psychiatrically disturbed inpatient children. Journal of Consulting and Clinical Psychology.

[B51-behavsci-16-00406] Keshoofy A., Pearl D. L., Lisnyj K., Thaivalappil A., Papadopoulos A. (2023). Risk and protective factors associated with hopelessness among Canadian postsecondary students. International Journal of Mental Health and Addiction.

[B52-behavsci-16-00406] King M., Jones L., Barnes K., Low J., Walker C., Wilkinson S., Tookman A. (2006). Measuring spiritual belief: Development and standardization of a beliefs and values scale. Psychological Medicine.

[B53-behavsci-16-00406] Kleiman E. M., Liu R. T. (2014). Prospective prediction of suicide in a nationally representative sample: Religious service attendance as a protective factor. British Journal of Psychiatry.

[B54-behavsci-16-00406] Klonsky E. D., Kotov R., Bakst S., Rabinowitz J., Bromet E. J. (2012). Hopelessness as a predictor of attempted suicide among first admission patients with psychosis: A 10-year cohort study. Suicide and Life-Threatening Behavior.

[B55-behavsci-16-00406] Kress V. E., Newgent R. A., Whitlock J., Mease L. (2015). Spirituality/religiosity, life satisfaction, and life meaning as protective factors for nonsuicidal self-injury in college students. Journal of College Counseling.

[B56-behavsci-16-00406] Kuo C. J., Tsai S. Y., Lo C. H., Wang Y. P., Chen C. C. (2005). Risk factors for completed suicide in schizophrenia. Journal of Clinical Psychiatry.

[B57-behavsci-16-00406] Lawrence R. E., Oquendo M. A., Stanley B. (2016). Religion and suicide risk: A systematic review. Archives of Suicide Research.

[B58-behavsci-16-00406] Lefevor G. T., McGraw J. S., Skidmore S. J. (2022a). Suicidal ideation among active and nonactive/former Latter-day Saint sexual minorities. Journal of Community Psychology.

[B59-behavsci-16-00406] Lefevor G. T., Scherer D., Larsen R. (2021). Religiousness, sexual orientation, and suicidal ideation: The role of religious conflict. Psychology of Sexual Orientation and Gender Diversity.

[B60-behavsci-16-00406] Lefevor G. T., Schow R. L., Beckstead A. L., Larsen R. (2022b). Religious and spiritual struggles and suicide risk among sexual minority individuals. Psychology of Sexual Orientation and Gender Diversity.

[B61-behavsci-16-00406] Luhmann M., Lucas R. E., Eid M., Diener E. (2013). The prospective effect of life satisfaction on life events. Social Psychological and Personality Science.

[B62-behavsci-16-00406] Lynch K. E., Gatsby E., Viernes B., Schliep K. C., Whitcomb B. W., Alba P. R., DuVall S. L., Blosnich J. R. (2020). Evaluation of suicide mortality among sexual minority US veterans from 2000 to 2017. JAMA Network Open.

[B63-behavsci-16-00406] Mahmoud J. S. R., Staten R. T., Hall L. A., Lennie T. A. (2012). The relationship among young adult college students’ depression, anxiety, stress, demographics, life satisfaction, and coping styles. Issues in Mental Health Nursing.

[B64-behavsci-16-00406] Mathy R. M., Cochran S. D., Olsen J., Mays V. M. (2011). The association between relationship markers of sexual orientation and suicide: Denmark, 1990–2001. Social Psychiatry and Psychiatric Epidemiology.

[B65-behavsci-16-00406] Matías R., Matud M. P. (2023). Mental symptoms, life satisfaction and sexual orientation: A gender analysis. Journal of Clinical Medicine.

[B66-behavsci-16-00406] Meyer I. (2003). Prejudice, social stress, and mental health in lesbian, gay, and bisexual populations: Conceptual issues and research evidence. Psychological Bulletin.

[B67-behavsci-16-00406] Meyer I. H., Russell S. T., Hammack P. L., Frost D. M., Wilson B. D. (2021). Minority stress, distress, and suicide attempts in three cohorts of sexual minority adults: A U.S. probability sample. PLoS ONE.

[B68-behavsci-16-00406] Mohr J., Fassinger R. (2006). Sexual orientation identity and romantic relationship quality in same-sex couples. Personality and Social Psychology Bulletin.

[B69-behavsci-16-00406] Morell-Mengual V., Gil-Llario M. D., Ballester-Arnal R., Salmerón-Sánchez P. (2017). Spanish adaptation and validation of the short internalized homonegativity scale (SIHS). Journal of Sex & Marital Therapy.

[B70-behavsci-16-00406] Mortier P., Cuijpers P., Kiekens G., Auerbach R. P., Demyttenaere K., Green J. G. (2017). The prevalence of suicidal thoughts and behaviours among college students: A meta-analysis. Psychological Medicine.

[B71-behavsci-16-00406] Mozumder M. K., Jasmine U. H., Haque M. A., Haque S. (2023). Mental health and suicide risk among homosexual males in Bangladesh. PLoS ONE.

[B72-behavsci-16-00406] Muehlenkamp J. J., Brausch A. M. (2012). Body image as a mediator of non-suicidal self-injury in adolescents. Journal of Adolescence.

[B73-behavsci-16-00406] Nahata L., Quinn G. P., Caltabellotta N. M., Tishelman A. C. (2017). Mental health concerns and insurance denials among transgender adolescents. LGBT Health.

[B74-behavsci-16-00406] Nock M. K., Banaji M. R. (2007). Prediction of suicide ideation and attempts among adoles cents using a brief performance-based test. Journal of Consulting and Clinical Psychology.

[B75-behavsci-16-00406] Nock M. K., Hwang I., Sampson N. A., Kessler R. C. (2010). Mental disorders, comorbidity and suicidal behavior: Results from the National Comorbidity Survey Replication. Molecular Psychiatry.

[B76-behavsci-16-00406] Nystedt T., Rosvall M., Lindström M. (2019). Sexual orientation, suicide ideation and suicide attempt: A population-based study. Psychiatry Research.

[B77-behavsci-16-00406] O’Brien E., Whitman K., Buerke M., Galfalvy H., Szanto K. (2023). Life-satisfaction, engagement, mindfulness, flourishing, and social support: Do they predict depression, suicide ideation, and history of suicide attempt in late life?. American Journal of Geriatric Psychiatry.

[B78-behavsci-16-00406] O’Reilly D., Rosato M. (2015). Religion and the risk of suicide: Longitudinal study of over 1 million people. British Journal of Psychiatry.

[B79-behavsci-16-00406] Pachankis J. E., Bränström R. (2018). Hidden from happiness: Structural stigma, sexual orientation concealment, and life satisfaction across 28 countries. Journal of Consulting and Clinical Psychology.

[B80-behavsci-16-00406] Pargament K. I., Koenig H. G., Perez L. M. (2000). The many methods of religious coping: Development and initial validation of the RCOPE. Journal of Clinical Psychology.

[B81-behavsci-16-00406] Park C. L., Waddington E., Abraham R. (2018). Different dimensions of religiousness/spirituality are associated with health behaviors in breast cancer survivors. Psycho-Oncology.

[B82-behavsci-16-00406] Pharr J. R., Batra K. (2024). Social–ecological determinants of suicidal ideation among sexual and gender minority adults: A cross-sectional study in the United States. Healthcare.

[B83-behavsci-16-00406] Plutchick R., Van Praga H. M., Conte H. R., Picard S. (1989). Correlates of suicide and violence risk1: The suicide risk measure. Comprehensive Psychiatry.

[B84-behavsci-16-00406] Pons D., Atienza F. L., Balaguer I., García-Merita M. L. (2000). Satisfaction with life scale: Analysis of factorial invariance for adolescents and elderly persons. Perceptual and Motor Skills.

[B85-behavsci-16-00406] Powdthavee N., Wooden M. (2015). Life satisfaction and sexual minorities: Evidence from Australia and the United Kingdom. Journal of Economic Behavior & Organization.

[B86-behavsci-16-00406] Ribeiro J. D., Huang X., Fox K. R., Franklin J. C. (2018). Depression and hopelessness as risk factors for suicide ideation, attempts and death: Meta-analysis of longitudinal studies. The British Journal of Psychiatry.

[B87-behavsci-16-00406] Ribeiro J. D., Pease J. L., Gutierrez P. M., Silva C., Bernert R. A., Rudd M. D., Joiner T. E. (2012). Sleep problems out perform depression and hopelessness as cross-sectional and longitudinal predictors of suicidal ideation and behavior in young adults in the military. Journal of Affective Disorders.

[B88-behavsci-16-00406] Riera-Serra P., Navarra-Ventura G., Castro A., Soler J., García-Campayo J., Gili M. (2024). Clinical predictors of suicidal ideation, suicide attempts and suicide death in depressive disorder: A systematic review and meta-analysis. European Archives of Psychiatry and Clinical Neuroscience.

[B89-behavsci-16-00406] Ringwald J., Wochnowski C., Bosse K., Giel E., Schäffeler N., Zipfel S., Teufel M. (2016). Psychological distress, anxiety, and depression of cancer-affected BRCA1/2 mutation carriers: A systematic review. Journal of Genetic Counseling.

[B90-behavsci-16-00406] Rosario M., Schrimshaw E. W., Hunter J. (2011). Different patterns of sexual identity development over time: Implications for the psychological adjustment of lesbian, gay and bisexual youths. Journal of Sex Research.

[B91-behavsci-16-00406] Rosario M., Schrimshaw E. W., Hunter J., Gwadz M. (2002). Gay-related stress and emotional distress among gay, lesbian and bisexual youths: A longitudinal examination. Journal of Consulting and Clinical Psychology.

[B92-behavsci-16-00406] Rotolone C., Martin G. (2012). Giving up self-injury: A comparison of everyday social and personal resources in past versus current self-injurers. Archives of Suicide Research.

[B93-behavsci-16-00406] Rönkä A. R., Taanila A., Koiranen M., Sunnari V., Rautio A. (2013). Associations of deliberate self-harm with loneliness, self-rated health and life satisfaction in adolescence: Northern Finland Birth Cohort 1986 study. International Journal of Circumpolar Health.

[B94-behavsci-16-00406] Rubio G., Montero I., Jáuregui J., Villanueva R., Casado M. A., Marín J. J., SantoDomingo J. (1998). Validación de la escala de riesgo suicida de Plutchik en población española. Archivos de Neurobiología: Revista de Psiquiatría y Disciplinas Afines.

[B95-behavsci-16-00406] Ryan P. C., Lowry N. J., Boudreaux E., Snyder D. J., Claassen C. A., Harrington C. J., Jobes D. A., Bridge J. A., Pao M., Horowitz L. M. (2024). Chronic pain, hopelessness, and suicide risk among adult medical inpatients. Journal of the Academy of Consultation-Liaison Psychiatry.

[B96-behavsci-16-00406] Salway T., Plöderl M., Liu J., Gustafson P. (2019). Effects of multiple forms of information bias on estimated prevalence of suicide attempts according to sexual orientation: An application of a bayesian misclassification correction method to data from a systematic review. American Journal of Epidemiology.

[B97-behavsci-16-00406] Sandler E. (2018). The ripple effect of suicide.

[B98-behavsci-16-00406] Sorbara J. C., Chiniara L. N., Thompson S., Palmert M. R. (2020). Mental health and timing of gender-affirming care. Pediatrics.

[B99-behavsci-16-00406] Stack S., Laubepin F. (2019). Religiousness as a predictor of suicide: An analysis of 162 European regions. Suicide and Life-Threatening Behavior.

[B100-behavsci-16-00406] St. John P. D., Mackenzie C., Menec V. (2015). Does life satisfaction predict five-year mortality in community-living older adults?. Aging & Mental Health.

[B101-behavsci-16-00406] Stuhlsatz G. L., Kavanaugh S. A., Taylor A. B., Neppl T. K., Lohman B. J. (2021). Spirituality and religious engagement, community involvement, outness, and family support: Influence on LGBT+ muslim well-being. Journal of Homosexuality.

[B102-behavsci-16-00406] Tedrus G. M. A. S., Souza D. C. M., Crepaldi C. R., Petrarca Y. M. (2023). Suicide risk in epilepsy: Clinical variables, psychiatric disorders, and social support. Revue Neurologique.

[B103-behavsci-16-00406] Thatcher W. G., Reininger B. M., Drane J. W. (2002). Using path analysis to examine adolescent suicide attempts, life satisfaction, and health risk behavior. Journal of School Health.

[B104-behavsci-16-00406] Toukhy N., Gvion Y., Barzilay S., Apter A., Haruvi-Catalan L., Bursztein-Lipsicas C., Shilian M., Mijiritsky O., Benaroya-Milshtein N., Fennig S., Hamdan S. (2024). Implicit identification with death, clinician evaluation and suicide ideation among adolescent psychiatric outpatients-the mediating role of depression. Archives of Suicide Research.

[B105-behavsci-16-00406] Trujillo M. A., Perrin P. B., Henry R. S., Rabinovitch A. E. (2020). Heterosexism and suicidal ideation: Racial differences in the impact of social support among sexual minority adults. Crisis: The Journal of Crisis Intervention and Suicide Prevention.

[B106-behavsci-16-00406] Vice Mental Health Guide (2015). LGBT mental health: Are we doing enough?.

[B107-behavsci-16-00406] Wang M., Wang B., Zuo Y., Tang Y., Zhang R., Lu Q. (2025). Relationship between life satisfaction and non-suicidal self-injury among adolescents: The chain mediating effect of depressive symptoms and cognitive dysfunction. Acta Psychologica.

[B108-behavsci-16-00406] WHO (2021a). One in 100 deaths is by suicide.

[B109-behavsci-16-00406] WHO (2021b). Suicide worldwide in 2019.

[B110-behavsci-16-00406] WHO (2025). Suicide.

[B111-behavsci-16-00406] Wolf J. K., Platt L. F. (2022). Religion and sexual identities. Current Opinion in Psychology.

[B112-behavsci-16-00406] Wu A., Wang J. Y., Jia C. X. (2015). Religion and completed suicide: A meta-analysis. PLoS ONE.

[B113-behavsci-16-00406] Wu L. (2011). Group integrative reminiscence therapy on self-esteem, life satisfaction and depressive symptoms in institutionalised older veterans. Journal of Clinical Nursing.

[B114-behavsci-16-00406] Yang Y., Zhang M., Kou Y. (2016). Self-compassion and life satisfaction: The mediating role of hope. Personality and Individual Differences.

[B115-behavsci-16-00406] Yildirim A., Akkus M., Asilar R. H. (2023). Hopelessness and life satisfaction in patients with serious mental disorders: A cross-sectional study. Northern Clinics of İstanbul.

